# Perineal and pelvic aggressive angiomyxoma: spontaneous regression after hormonal replacement therapy withdrawal assessed by 3T magnetic resonance

**DOI:** 10.1259/bjrcr.20150110

**Published:** 2015-07-07

**Authors:** R Argiró, B Sacconi, A Iannarelli, P Collini, M Bezzi

**Affiliations:** ^1^ Department of Radiological, Oncological and Anatomopathological Sciences—Radiology—Sapienza, University of Rome, Italy; ^2^ Department of Pathology, Istituto Nazionale dei Tumori, Milano Italy

## Abstract

Aggressive angiomyxoma (AA) is a rare mesenchymal tumour which mainly arises in the soft tissue of the pelvis and perineum in women of reproductive age. AA usually shows an aggressive behaviour, with a high rate of incomplete surgical excision and post-surgical recurrence. Most cases of AA exhibit oestrogen and/or progesterone receptors; in these cases, maintenance of a hypo-oestrogenic state can be helpful in the medical management of this tumour. We describe a case of spontaneous reduction in size of an AA during a 6-month period of hormonal replacement therapy withdrawal, assessed by MRI using a 3T magnet.

## Summary

Aggressive angiomyxoma (AA) is a rare mesenchymal tumour mainly arising in the soft tissue of the pelvis and perineum in women of reproductive age.^1^ To date, fewer than 350 cases have been reported in the English-language literature.

The locally aggressive behaviour of this tumour produces a large, infiltrative mass lesion, and complete surgical excision is often difficult or impossible with high rates of recurrence. Most cases of AA exhibit positive immunohistochemical staining with oestrogen receptors (ER) and/or progesterone receptors (PR),^2^ including the case we describe, which was ER positive. Maintenance of a hypo-oestrogenic state can be helpful in the medical management of this tumour.^3,4^

We describe a case of spontaneous reduction in size of an AA assessed by MRI during a 6-month period of hormonal replacement therapy (HRT) withdrawal.

## Case report

We present a case of spontaneous reduction in size of a perineal and pelvic AA in a 50-year-old female patient observed during a 6-month period of HRT withdrawal and well assessed using 3T MRI. The patient was referred to our hospital by the gynaecologist to undergo a pelvic MRI because of worsening back pain and vague pelvic discomfort.

She previously underwent a radical hysteroannessectomy (for uterine fibromas and an endometrial/mucinous cyst on the left ovary) and a transurethral resection of the bladder (for a low-grade papilloma), 5 and 2 years earlier, respectively. After the hysteroannessectomy, the patient was treated with HRT (with a transdermal gel formulation for the first year and then oral tablets at a daily dose of 1 mg).

MRI protocol, performed with a 3T magnet (Verio, Siemens AG, Erlangen, Germany) using 8-channel surface coil, included *T*_2_ weighted images on three planes: axial fat-suppressed *T*_2_ weighted sequences, axial and coronal fat-suppressed *T*_1_ weighted sequences before and after contrast media injection (1 ml kg^–1^ of gadobenate dimeglumine, MultiHance, Bracco, Milan, Italy) and diffusion weighted sequences (DWI).

**Figure 1.**
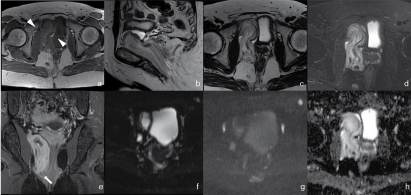
(a) Axial unenhanced *T*_1_ weighted MRI shows a pelvic mass primarily isointense to muscle (arrowheads) with few areas of high signal intensity. (b) Sagittal *T*_2_ weighted MRI displays major high signal intensity interspersed with swirled or layered strands of lower signal intensity. (c,d) Axial *T*_2_ weighted and fat-suppressed *T*_2_ weighted images show the same swirling appearance with lack of major adipose components. Marked compression of the bladder is notable and mild displacing of vaginal and anal canal is shown. (e) Coronal post-contrast *T*_1_ weighted MRI with fat suppression reveals the swirling pattern of remarkable enhancement within the tumour and infiltration of right elevator ani muscle (arrow). No signs of internal obturator muscle infiltration were observable. (f,g) Diffusion weighted imaging sequences (B0–B1000) demonstrate heterogeneous high signal. (h) ADC shows quite homogeneous hyperintense signal on ADC map. ADC, apparent diffusion coefficient.

MRI revealed a well-defined, 9 × 5-cm mass lesion arising from the right perianal fat tissue. The lesion displaced contralaterally the anal canal and the vagina, whereas the bladder was markedly compressed. Sagittal *T*_2_ weighted sequence showed a “finger-like” extension of the lesion into the right ischiorectal fossa; signs of infiltration of the right elevator ani muscle were also observed. The lesion was quite homogeneously isointense in comparison to muscle on *T*_1_ weighted images. On *T*_2_ weighted and fat-suppressed *T*_2_ weighted images, the lesion mainly showed high signal intensity, with layered wave-like strands of lower signal intensity. The mass markedly enhanced after contrast media administration, with a “swirling” pattern. DWI showed heterogeneous high signal intensity on B0 and B1000 sequences; apparent diffusion coefficient (ADC) mapping showed a high value in the tumour (Figure 1). Based on peculiar localization and MRI findings, radiologists suggested the diagnosis of AA.

**Figure 2.**
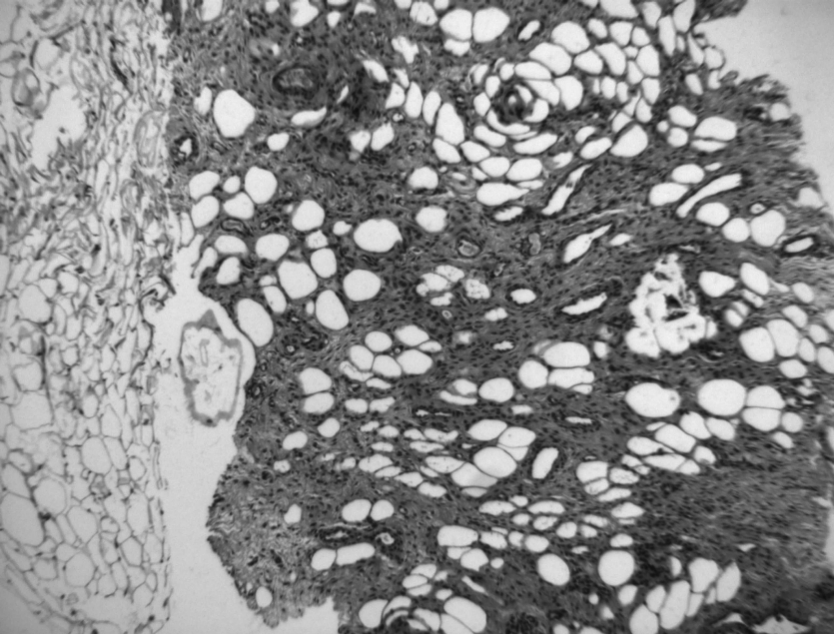
Locally infiltrative growth of bland fibro-myofibroblastic cells with characteristic thick-walled blood vessels and a focally myxoid stroma into adipose tissue (HE, 5×).

A CT-guided biopsy with a Tru-Cut 16G needle with a perineal approach was performed; pathologists definitively confirmed the diagnosis of AA. The tumour histologically consisted of spindle cells in a myxoid stroma, containing a mixture of thick- and thin-walled blood vessels with interposition of normal fat tissue (Figure 2). Immunohistochemically, the tumour cells exhibited diffuse nuclear positivity with ER.

Radical surgery was not thought to be feasible; the therapeutic decision, on the basis of hyperexpression of ER, was to suspend HRT in order to reduce the oestrogen stimulation and hence cell proliferation. A short-term MRI follow-up was planned.

**Figure 3.**
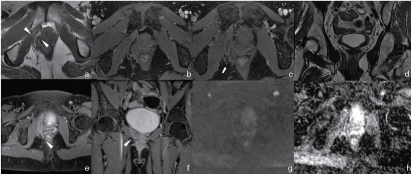
6-month MRI control scan after hormonal replacement therapy withdrawal. (a) Axial *T*_2_ weighted image shows a great reduction in size of the tumour (arrowheads). (b,c) Axial fat-suppressed *T*_2_ weighted images show loss of main hyperintensity and “finger-like” extension still notable in the right ischiorectal fossa (arrow). (d) Coronal *T*_2_ weighted image shows main loss of swirling appearance. (e,f) Axial and coronal post-contrast *T*_1_ weighted images display decrease in internal vascularization. Bladder compression is no longer evident. Signs of elevator ani muscle infiltration are still notable (arrowhead). (g) Diffusion weighted imaging (B1000) shows a soft hyperintense signal. (h) On apparent diffusion coefficient map, the lesions confirm its quite homogeneous hyperintensity.

6 months later, the patient came back to our department owing to regression of symptoms and underwent a new MRI scan that revealed a marked reduction in size of the tumour (about 60–70% in volume); bladder compression and surrounding structures’ infiltration were also reduced. Post-contrast fat-suppressed *T*_1_ weighted images showed a reduction in the lesion’s vascularization. On DWI sequences, AA remained hyperintense on both B0 and B1000 images and ADC map (Figure 3).

## Discussion

AA is an uncommon mesenchymal tumour mainly occurring in the genital and perineal area of female patients in the third to fifth decades.^1,2^ So far, only a few cases of AA have been reported in males. Most tumours are slow-growing large lesions (usually larger than 5–6 cm). Signs and symptoms at presentation may include discomfort from the mass or pressure effects on adjacent pelvic organs.^1^

Although surgical excision is usually performed with wide tumour-free margins, most patients experience local recurrences because of the locally infiltrative nature of this tumour (approximately 70% after a period of 2 years);^5^ only in occasional cases have metastases been described.^6^

In our case, surgery was not performed. The patient refused consent for a radical surgery because the internal elevator ani muscle required excision; on the other hand, preserving this muscle would have increased the risk of local recurrence. HRT withdrawal was considered a potential therapeutic option, considering the tumour ER expression. The role of oestrogens in cancer genesis has already been demonstrated for a few tumours (such as breast cancer); the most widely accepted theory is that estradiol, acting through oestrogen receptor-α, stimulates cell proliferation and leads to mutations arising from replicative errors during premitotic DNA synthesis.^2–4^ A similar mechanism can be hypothesized in AA; the finding of positive immunohistochemical staining with ER and/or PR thus provides a rationale for the medical management of AA, which consists of inducing an hypo-oestrogenic state.^2,7^ This can be obtained by using gonadotropin releasing hormone (GnRH) agonists. GnRH agonists usually induce an initial stimulation of the pituitary gland with increased production of follicle-stimulating hormone (FSH) and luteinizing hormone (LH). However, further administration results in desensitizing of the pituitary to GnRH with a subsequently decreased production of FSH and LH, resulting in a hypo-oestrogenic state. In our case, since HRT withdrawal allowed a marked reduction in size of the tumour, we can hypothesize that a further improvement in disease control may have been obtained by using GnRH agonists.^3,4^

AA can show typical MRI features: images usually show a perineal mass lesion displacing rather than invading adjacent organs (such as urethra, vagina, anal sphincter, rectum and surrounding fat) and frequently crossing the pelvic diaphragm.^8–10^

The tumour is usually isointense compared with muscle on *T*_1_ weighted images, hyperintense on *T*_2_ weighted images and avidly enhances after contrast media administration, with typical swirling and layering internal patterns. The hyperintensity on *T*_2_ weighted images is most likely owing to the high water and myxoid matrix content of the tumour, while the avid enhancement reflects its high vascularity.^8–10^ The presence of swirling or layering strands in the tumour after contrast media administration can be considered a distinctive diagnostic feature (occurring in about 83% of patients); these strands usually present lower signal intensity in comparison with the remaining tumour on *T*_2_ weighted and post-contrast *T*_1_ weighted images.^9^ AA can show high signal intensity in both DWI sequence and ADC map, as described recently.^11^ The differential diagnosis of AA includes angiomyofibroblastoma, superficial angiomyxoma, fibroepithelial stromal polyps, myxoid lipomatous tumours and myxoid leiomyoma.

Although AA is an uncommon tumour, MRI features have been described by several authors.^8–10^ However, to our knowledge, this is the first case report assessing the reduction in size of an AA during medical treatment using MRI; furthermore, this high tumour response was observed in a female patient not on medical therapy with GnRH agonists, but undergoing only HRT withdrawal.

## Learning Points

1. AA is a rare mesenchymal tumour mainly arising in the soft tissue of the pelvis and perineum in women of reproductive age.
2. AA shows a locally aggressive behaviour with the evidence of focal sign of infiltration and compression of surrounding structures.
3. Complete excision is often difficult or impossible and recurrences after surgery are frequent.
4. AA often presents as a well-defined mass. On *T*_1_ images, AA is isointense to muscle and on *T*_2_ weighted and fat-suppressed *T*_2_ weighted images, it appears globally hyperintense.
5. The presence of swirling or layering strands in the tumour on *T*_1_ weighted images after contrast media administration can be considered a distinctive diagnostic feature (occurring in about 83% of patients).
6. DWI showed heterogeneous high signal intensity on B0 and B1000 sequences; ADC mapping showed a high value of the tumour.
7. Medical management can be employed due to the presence of ER receptors, inducing a hypo-oestrogenic state.
